# Iron deficiency in acute coronary syndromes: prevalence and prognostic impact

**DOI:** 10.1097/j.pbj.0000000000000278

**Published:** 2025-01-08

**Authors:** Ana Fátima Esteves, Sara Gonçalves, Tatiana Duarte, Joana Ferreira, Rui Coelho, Jéni Quintal, Catarina Pohle, Nuno Fonseca, Rui Caria

**Affiliations:** Rua Camilo Castelo Branco, Cardiology Department, São Bernardo Hospital, Setúbal Hospital Centre, Setúbal, Portugal

**Keywords:** acute coronary syndrome, iron deficiency, heart failure

## Abstract

**Introduction and Objectives:**

Iron deficiency (ID) is a well-known prognostic marker in heart failure (HF), independent of anemia. However, its impact in acute coronary syndromes (ACSs) is not well established.

**Methods:**

This is a retrospective analysis of patients admitted with ACS from January to December 2019. The population was evaluated according to basal characteristics and divided in two groups, with or without ID, compared according to several variables, namely demographic, index hospitalization, comorbidities, analytical and echocardiographic. We determined their predictive value on the occurrence of hemorrhage or need for red blood cell transfusion, all-cause hospitalizations, and all-cause death.

**Results:**

We included 287 patients, with median age 66 (interquartile range [IQR] 21) years and 72% male. ID was present in 48% of patients. Most presented with ST-elevation myocardial infarction (STEMI) (57%), and 13% were admitted in Killip–Kimball class III or IV. Almost 40% had a left ventricle ejection fraction < 50% on admission. Patients with non–ST-elevation myocardial infarction presented more frequently with ID, compared with patients with STEMI. Of note, 33% of patients with ID had concomitant anemia. During a median follow-up of 28 (IQR 7) months, 10% had an urgent care admission for HF, 7% were hospitalized for HF, and 18% died. There was a significantly higher rate of emergency department admissions for HF in the group with ID. Age, anemia, and NT-proBNP levels predicted the occurrence of all-cause death, with ID having no impact on none of the evaluated events.

**Conclusion:**

ID was prevalent in patients with ACS, affecting almost half of the population. In the acute setting of an ACS, anemia seems to have a greater impact on mortality and the role of ID in promoting HF needs further investigation.

## Abstract 2

Supplemental Digital Content is Available in the Text.

## Introduction

Iron is a fundamental element for human life, being crucial for many physiological and cellular processes and involved in erythropoiesis, oxygen transport, immune response, and mitochondrial metabolism.^1–3^

Iron deficiency (ID) is a very prevalent condition, which is present in up to one-third of the world's population and affects predominantly children, adolescents, elderly people, and premenopausal women, causing a great burden of disease, independent of the presence of anemia.^4,5^ It can be subdivided into absolute and functional ID, the former occurring when iron stores are reduced and insufficient to meet the body's needs and the latter being associated with inflammation, which increases hepcidin concentrations and impairs iron absorption and its mobilization to the plasma, affecting erythropoiesis despite adequate iron stores.^6,7^

Disorders of iron metabolism are frequent in patients with cardiovascular (CV) disease.^8,9^ The role of ID in heart failure (HF) is nowadays well substantiated by a number of studies and clinical trials, which have shown its extremely high prevalence, being a common comorbidity present in up to 55% of patients with chronic HF and that could reach up to 80% of those with acute HF.^5,10^ In addition, ID has a negative prognostic impact on patients with HF, causing a reduction in exercise capacity, physical well-being, and quality of life.^11–13^ In addition, it is independently associated with recurrent HF hospitalizations and high CV and all-cause mortality.^10,14,15^ Of note, ID can have a negative impact on mortality independent of the presence of anemia.^13,16^

As such, current European Society of Cardiology (ESC) HF guidelines attributed a class IC recommendation for the screening of ID in all patients with HF, using serum ferritin and transferrin saturation (TSAT). In patients with HF, ID is defined as serum ferritin level either <100 ng/mL or 100–299 ng/mL with TSAT <20%, denoting absolute and relative ID, respectively.^17,18^ In fact, in inflammation disorders such as infection, cancer, liver disease, and CV diseases such as HF and acute coronary syndromes (ACSs), there is increased tissue ferritin expression that causes its high concentration in peripheral blood, illustrating the necessity for higher cutoff values for the definition of ID in these settings.^18,19^

ACS is one of the current major causes of morbidity and mortality around the globe.^20^ Information regarding the impact of ID on these patients is still scarce and its clinical relevance is still unclear, but data have been emerging in the past few years, showing that iron metabolism disorders and ID are prevalent in patients with ACS and are associated with adverse events and a worse prognosis.^1,21–23^ A recent meta-analysis that assessed the long-term prognostic impact of ID on ACS found a worse prognosis compared with patients without ID, regarding all-cause mortality or a combined end point of nonfatal myocardial infarction (MI) and CV mortality; exercise capacity and quality of life were also significantly reduced.^24^ However, it is important to note the heterogeneity across different studies assessed in this analysis.

## Objectives

Our purpose was to evaluate the prevalence of ID in patients with ACS and its prognostic impact during follow-up.

## Methods

This was a retrospective, single-center, longitudinal analysis that included all patients admitted to a coronary care unit/cardiology ward with the diagnosis of ACS between January 2019 and December 2019. This center was a hospital with 24 hours/7 days a week availability of primary percutaneous coronary intervention but without cardiac surgery on-site. Intra-aortic balloon pump (IABP) was the only mechanical circulatory support device available.

Patients with ST-elevation myocardial infarction (STEMI), non–ST-elevation MI (NSTEMI), and unstable angina (UA), defined according to current ESC guidelines,^20,25^ were included. We excluded patients with inexistent measurements of ferritin or TSAT during index hospitalization. Patients who were transferred to other institutions after initial admission were still included in the analysis if they met the inclusion criteria and had available ferritin and TSAT measurements.

Patient data were collected, including demographic characteristics, laboratory values, presence of comorbidities, medical history, concomitant medication, variables related to the index hospitalization (length of hospital stay, type of ACS, number of diseased vessels, left main disease, therapeutic approach regarding ACS, Killip–Kimball [KK] class on admission), events during index hospitalization (atrial and ventricular tachyarrhythmias, high-grade/complete atrioventricular block, acute HF, need for vasopressor/inotropic drugs or IABP, acute kidney injury, repeated coronary angiography, hemorrhage, red blood cell transfusion, ferric carboxymaltose administration, infections, cerebrovascular events, and death), echocardiographic variables on index hospitalization (left ventricle ejection fraction [LVEF], LV dimensions, tricuspid annular plane systolic excursion, and significant valve stenosis or regurgitation, assessed at admission with Vivid iq GE Healthcare according to specific guidelines),^26,27^ medication at discharge, and adverse events during follow-up (stroke/transient ischemic attack [TIA], ACS, hemorrhage defined by Bleeding Academic Research Consortium [BARC] 3 or higher hemorrhagic event^28^ or need for red blood cell [RBC] transfusion, emergency department admissions due to HF, hospitalizations due to HF, all-cause hospitalizations, CV death, and all-cause death). Of note, CV risk factors were defined by one or more of the following: previous description on initial clinical assessment; previous use of specialized medication; specific findings on clinical or laboratory assessment during hospitalization; patient statement (namely in case of smoking status). Alcohol use disorder was defined as a problematic pattern of alcohol use leading to clinically significant impairment, according to the Diagnostic and Statistical Manual of Mental Disorders, 5th Edition, Text Revision (DSM-5-TR),^29^ and gastrointestinal disease encompassed previous description on initial clinical assessment or specific finding during hospitalization of peptic ulcer disease, pancreatitis, cholestatic disorders, or chronic hepatic disease/cirrhosis. The term neoplasia referred to active neoplasia (currently under treatment) or neoplasia diagnosed and/or treated in the past 5 years.

Peripheral venous blood samples were collected from all patients on admission and during index hospitalization as deemed necessary. Iron status was measured in all patients in the first 24 hours of admission through assessment of serum iron (mc/dL), serum ferritin (ng/mL), total iron binding capacity (TIBC, mc/dL), and TSAT (in percentage).

Anemia was defined as hemoglobin (Hb) < 13.0 g/dL in men and <12.0 g/dL in women, according to the World Health Organization (WHO) definition.^30^

### Statistical analysis

The data obtained were entered in Microsoft Excel and subsequently analyzed in R (Bell Laboratories, New Jersey), version 4.2.1.

The population was evaluated according to basal characteristics and divided into two groups, with or without ID, as defined above (i.e., per the ESC HF guidelines). The groups were compared according to the variables described above, and their predictive value on the occurrence of hemorrhage or need for RBC transfusion, all-cause hospitalizations, and all-cause death was determined.

A Shapiro–Wilk test was performed to test for the normality of continuous variables. In the presence of normality, data are expressed as mean and standard deviation (SD) and, in its absence, as median and interquartile range (IQR). Data are presented as frequencies and percentages for categorical variables. Differences between groups were assessed with the use of the Pearson χ^2^ test, Welch two-sample *t* test, Wilcoxon rank-sum test with continuity correction (for non-normal continuous data), and Fisher exact test, as appropriate.

Cox regression analysis was used to calculate the odds ratios (ORs) and 95% confidence intervals (CIs). Kaplan–Meier curves were created to demonstrate the impact of ID on adverse events during follow-up and cumulative survival. Univariate and multivariate Cox proportional hazard regression models were used to assess the predictive value of ID on all-cause mortality. A value of *P* < .05 was considered statistically significant.

## Results

From January to December 2019, 359 patients were admitted to the coronary care unit/cardiology ward with a diagnosis of ACS. Of these, 72 patients were excluded for lack of determined TSAT and ferritin, and thus, a total of 287 were included in the study.

### Baseline population characteristics

The baseline characteristics of the population studied are presented in Table 1. The median age was 66 (IQR 21) years, 72% of male sex. ID was present in 48% of patients. CV risk factors were frequent, especially arterial hypertension and dyslipidemia, and 22% of patients had a history of coronary artery disease (CAD), with 19% having suffered a previous MI. Most of the patients presented with STEMI (57%), 35% had 3-vessel coronary disease and 2% had isolated left main disease, with 13% being admitted in KK class III or IV. All but one patient underwent coronary angiography, and most were treated using percutaneous coronary intervention, with 10% being treated conservatively. Thirty-eight percent of patients had a LVEF < 50% on admission.

**Table 1:** Baseline patient characteristics.

	Total (n = 287)	ID (n = 137)	No ID (n = 150)	*P*
Demographic				
Age in years, median (IQR)	66.0 (21.0)	65.0 (20.0)	67.0 (21.0)	.789
Male, n (%)	206 (71.8)	80 (58.4)	126 (84.0)	**<.001**
CV risk factors				
Hypertension, n (%)	208 (72.5)	102 (74.5)	106 (70.7)	.559
Diabetes mellitus, n (%)	94 (32.8)	53 (38.7)	41 (27.3)	.055
Dyslipidemia, n (%)	237 (82.6)	118 (86.1)	119 (79.3)	.174
Smoking, n (%)	166 (57.8)	76 (55.5)	90 (60.0)	.474
Medical history/comorbidities				
Coronary artery disease, n (%)	63 (22.0)	35 (25.5)	28 (18.7)	.206
Previous PCI, n (%)	48 (16.7)	26 (19.0)	22 (14.7)	.413
Previous CABG, n (%)	13 (4.5)	6 (4.4)	7 (4.7)	1.000
Peripheral artery disease, n (%)	14 (4.9)	8 (5.8)	6 (4.0)	.654
Stroke/TIA, n (%)	21 (7.3)	13 (9.5)	8 (5.3)	.261
Heart failure, n (%)	4 (1.4)	4 (2.9)	0 (0.0)	.109
Atrial fibrillation, n (%)	19 (6.6)	6 (4.4)	13 (8.7)	.222
Chronic kidney disease, n (%)	40 (13.9)	20 (14.9)	20 (13.3)	.830
COPD, n (%)	7 (2.4)	3 (2.2)	4 (2.7)	1.000
Alcohol use disorder, n (%)	12 (4.2)	5 (3.6)	7 (4.7)	.893
Anemia, n (%)	91 (31.7)	45 (32.8)	46 (30.7)	.788
Neoplasia, n (%)	28 (9.8)	11 (8.0)	17 (11.3)	.547
Gastrointestinal disease, n (%)	18 (6.3)	10 (7.3)	8 (5.3)	.658
Previous medication				
Antiplatelets, n (%)	76 (26.5)	44 (32.1)	32 (21.3)	.053
Anticoagulants, n (%)	16 (5.6)	7 (5.1)	9 (6.0)	.943
ACEi, n (%)	62 (21.6)	32 (24.4)	30 (21.1)	.613
ARB, n (%)	68 (23.7)	38 (29.0)	30 (21.1)	.173
ARNI, n (%)	1 (0.3)	1 (0.8)	0 (0.0)	.968
Beta-blocker, n (%)	71 (24.7)	43 (32.8)	28 (19.7)	**.020**
MRA, n (%)	7 (2.4)	4 (3.1)	3 (2.1)	.914
Diuretic, n (%)	68 (23.7)	38 (29.0)	30 (21.1)	.173
SGLT2i, n (%)	10 (3.5)	5 (3.8)	5 (3.5)	1.000
Oral antidiabetic, n (%)	72 (25.1)	41 (31.3)	31 (21.7)	.095
Insulin, n (%)	18 (6.3)	16 (12.2)	2 (1.4)	**<.001**
Statin, n (%)	109 (38.0)	59 (45.0)	50 (35.0)	.115
Clinical presentation and treatment				
LOS in days (median, IQR)	5.0 (5.0)	6.0 (5.0)	5.0 (6.0)	.169
STEMI, n (%)	163 (56.8)	67 (48.9)	96 (64.0)	**.014**
NSTEMI, n (%)	119 (41.5)	67 (48.9)	52 (34.7)	**.020**
UA, n (%)	5 (1.7)	3 (2.2)	2 (1.3)	.919
1-vessel CAD, n (%)	87 (30.3)	41 (29.9)	46 (30.9)	.964
2-vessel CAD, n (%)	92 (32.1)	37 (27.0)	55 (36.9)	.096
3-vessel CAD, n (%)	101 (35.2)	54 (39.4)	47 (31.5)	.205
KK I, n (%)	241 (84.0)	114 (83.2)	127 (84.7)	.737
KK II, n (%)	10 (3.5)	5 (3.6)	5 (3.3)	1.000
KK III or IV, n (%)	36 (12.5)	18 (13.1)	18 (12.0)	.910
PCI, n (%)	238 (82.9)	109 (79.6)	129 (86.0)	.197
CABG, n (%)	21 (7.3)	10 (7.3)	11 (7.4)	1.000
Conservative treatment, n (%)	28 (9.8)	18 (13.1)	10 (6.7)	.104
Anemia, n (%)	91 (31.7)	45 (32.8)	46 (30.7)	.788
Iron deficiency, n (%)	137 (47.7)	137 (47.7)	—	—
Absolute iron deficiency, n (%)	67 (23.3)	67 (23.3)	—	—
Functional iron deficiency, n (%)	70 (24.4)	70 (24.4)	—	—

Bold denotes significant values (*p* < 0.05). ACEi, angiotensin-converting enzyme inhibitor; ARB, angiotensin II receptor blocker; ARNI, angiotensin receptor-neprilysin inhibitor; CABG, coronary artery bypass grafting; CAD, coronary artery disease; COPD, chronic obstructive pulmonary disease; ID, iron deficiency; IQR, interquartile range; KK, Killip–Kimball; LOS, length of hospital stay; MRA, mineralocorticoid receptor antagonist; NSTEMI, non–ST-elevation myocardial infarction; PCI, percutaneous coronary intervention; SGLT2i, sodium-glucose cotransporter-2 inhibitor; STEMI, ST-elevation myocardial infarction; TIA, transient ischemic attack; UA, unstable angina.

There were significant differences between groups with and without ID regarding sex, with a higher percentage of women with ID. Patients with ID were more frequently treated with beta-blockers and insulin. STEMI was more common in non-ID patients.

There were no significant differences regarding presence of anemia. Of note, 33% of patients with ID had concomitant anemia.

### Laboratory and echocardiographic data

Regarding laboratory data on admission (Table 2), there were no significant differences between groups with the exception of hemoglobin and serum iron values, which, as expected, were higher in patients without ID.

**Table 2:** Laboratory and echocardiographic data.

	ID (n = 137)	No ID (n = 150)	*P*
Laboratory values on admission			
Hemoglobin in g/dL, median (IQR)	13.10 (2.1)	13.80 (2.2)	**.031**
Serum iron in mc/dL, median (IQR)	48.0 (32.0)	69.5 (42.0)	**<.001**
NT-proBNP in pg/mL, median (IQR)	1515.0 (2980.0)	1045.0 (2910.0)	.154
Laboratory values during index hospitalization			
Peak hs-TnI in pg/mL, median (IQR)	13410.0 (24200.0)	15030.0 (23700.0)	.626
Peak sCr in mg/dL, median (IQR)	1.01 (0.6)	1.04 (0.5)	.330
Echocardiographic data on admission			
LVEF <50%, n (%)	53 (38.7)	56 (37.6)	.944
Left ventricular hypertrophy, n (%)	9 (6.6)	1 (0.7)	**.017**
RV dysfunction, n (%)	14 (12.2)	18 (12.1)	.756
Moderate or severe AS, n (%)	12 (8.8)	9 (6.0)	.513
Moderate or severe MR, n (%)	13 (9.5)	10 (6.7)	.519

Bold denotes significant values (*p* < 0.05). AS, aortic stenosis; hs-TnI, high-sensitivity troponin I; ID, iron deficiency; IQR, interquartile range; LVEF, left ventricle ejection fraction; MR, mitral regurgitation; NT-proBNP, N-terminal-proB-type natriuretic peptide; RV, right ventricle; sCr, serum creatinine.

In what concerns echocardiographic parameters on admission, there was a higher percentage of left ventricular hypertrophy in the ID patient group and there were no significant differences regarding LVEF < 50%.

### Events during index hospitalization and medication at discharge

Comparison of events during index hospitalization between groups is presented in Table 3. Of note, in case of patient transfer to another institution (namely to undergo coronary artery bypass grafting), the events sustained during the remainder of the hospitalization were included in the analysis.

**Table 3:** Events during index hospitalization and medication at discharge.

	ID (n = 137)	No ID (n = 150)	*P*
Index hospitalization events			
Ventricular tachycardia, n (%)	21 (15.3)	22 (14.7)	1.000
De novo atrial fibrillation, n (%)	17 (12.4)	18 (12.0)	1.000
High-grade/complete AV block, n (%)*	8 (5.8)	13 (8.7)	.479
Temporary transvenous pacemaker, n (%)	2 (1.5)	6 (4.0)	.344
Acute heart failure, n (%)	19 (13.9)	18 (12.0)	.768
Vasopressor and inotropic drugs, n (%)*	11 (8.0)	12 (8.1)	1.000
Levosimendan, n (%)*	2 (1.5)	2 (1.3)	1.000
IABP, n (%)	0 (0.0)	1 (0.7)	1.000
Acute kidney injury, n (%)	40 (29.2)	36 (24.0)	.388
Repeated coronary angiography, n (%)*	23 (16.8)	22 (14.7)	.740
Hemorrhage, n (%)	10 (7.3)	10 (6.7)	1.000
RBC transfusion, n (%)	6 (4.4)	4 (2.7)	.640
Ferric carboxymaltose administration, n (%)	7 (5.1)	1 (0.7)	.054
Infection, n (%)	31 (22.6)	26 (17.3)	.330
Stroke/TIA, n (%)	1 (0.7)	2 (1.3)	1.000
CV death, n (%)	3 (2.2)	7 (4.7)	.412
All-cause death, n (%)	3 (2.2)	9 (6.0)	.188
Medication at discharge			
Acetylsalicylic acid, n (%)	127 (92.7)	142 (94.7)	.658
Ticagrelor, n (%)	80 (58.4)	96 (64.9)	.317
Clopidogrel, n (%)	51 (37.2)	45 (30.4)	.275
Anticoagulants, n (%)	24 (17.5)	25 (16.8)	.993
DOAC, n (%)	18 (13.2)	18 (12.1)	.909
VKA, n (%)	4 (2.9)	7 (4.7)	.645
DAPT duration in months, median (IQR)	17.0 (14.0)	16.0 (13.0)	.424
Oral iron, n (%)	4 (4.1)	2 (1.9)	.608

* Missing values.

AV, atrioventricular; CV, cardiovascular; DAPT, dual antiplatelet therapy; DOAC, direct-acting oral anticoagulants; IABP, intra-aortic balloon pump; ID, iron deficiency; IQR, interquartile range; RBC, red blood cell; TIA, transient ischemic attack; VKA, vitamin K antagonist.

There were no significant differences between groups.

### Adverse events during follow-up

During a median follow-up of 28 (IQR 10 in patients with ID; IQR 7 in non-ID patients) months, 8 patients (3%) had a stroke/TIA, 49 (17%) a BARC 3 or higher hemorrhagic event/need for RBC transfusion, and 21 (7%) an ACS. Furthermore, 29 patients (10%) had an urgent care admission for HF, 20 (7%) were hospitalized due to HF, and 51 (18%) died.

There were significant differences between groups in the rate of emergency department admissions for HF, higher in the group with ID (15% versus 7%). There were no differences in the rates of ACS, stroke/TIA, all-cause hospitalizations, or mortality (Table 4).

**Table 4:** Adverse events during follow-up.

	ID (n=137)	No ID (n=150)	*P*-value
Adverse events during follow-up			
Stroke/TIA, n (%)	5 (3.8)	3 (2.2)	.680
ACS, n (%)	11 (8.3)	10 (7.2)	.930
Hemorrhage (BARC ≥ 3) or need for RBC transfusion, n (%)	28 (21.2)	21 (15.6)	.300
Emergency department admissions for HF, n (%)	20 (15.0)	9 (6.6)	**.040**
Hospitalizations for HF, n (%)	14 (10.5)	6 (4.3)	.087
All-cause hospitalizations, n (%)	35 (26.3)	28 (20.3)	.303
CV death, n (%)	8 (6.1)	11 (7.6)	.780
All-cause death, n (%)	21 (15.3)	30 (20.1)	.365

Bold denotes significant values (*p* < 0.05). ACS, acute coronary syndrome; BARC, Bleeding Academic Research Consortium; CV, cardiovascular; HF, heart failure; ID, iron deficiency; RBC, red blood cell; TIA, transient ischemic attack.

### Assessment of prognosis

We analyzed the role of different factors in patients' prognosis, namely on the occurrence of hemorrhage or need for RBC transfusion, all-cause hospitalizations, and all-cause death, and designed models for the prediction of these adverse events based on logistic regression models (which are detailed in the supplementary file, http://links.lww.com/PBJ/A41).

Age, presence of anemia, and NT-proBNP levels were the only variables that predicted, to some extent, the occurrence of all-cause death, with ID having no impact on none of the evaluated events.

**Figure 1.**
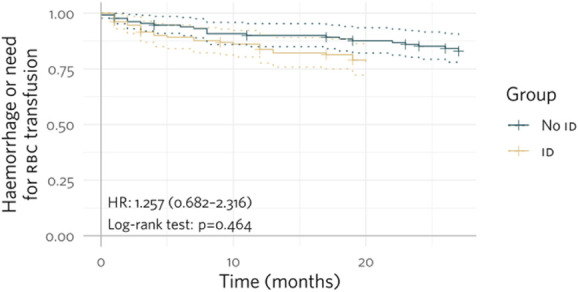
Kaplan–Meier curves for event-free rates of the occurrence of hemorrhage or need for RBC transfusion in patients with or without ID.

**Figure 2.**
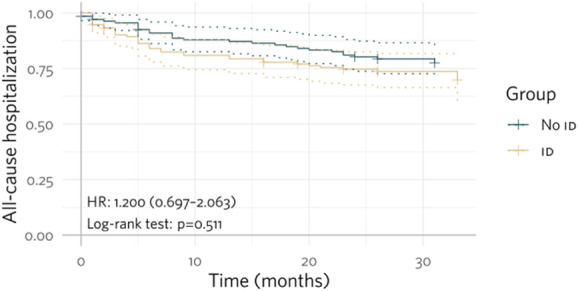
Kaplan–Meier curves for event-free rates of all-cause hospitalizations in patients with or without ID.

**Figure 3.**
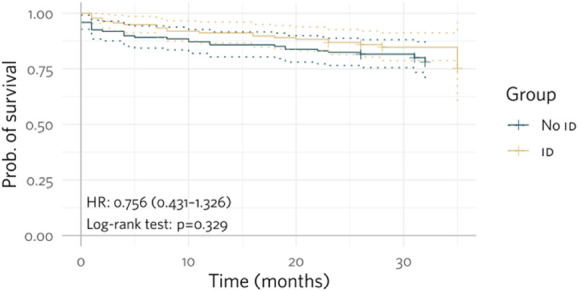
Kaplan–Meier curves for event-free rates of all-cause death in patients with or without ID.

Event-free Kaplan–Meier curves regarding the occurrence of hemorrhage or need for RBC transfusion, all-cause hospitalizations, and all-cause death show the difference between the groups with or without ID (Figs. 1–3).

## Discussion

In this retrospective cohort of patients hospitalized for ACS, we demonstrate that ID is very common in patients with ACS—nearly half of the patients present this condition. However, this prevalence of ID is somewhat lower than what has been described in studies examining the prevalence and impact of ID in acute HF.^5,10^ Regarding some cohorts of patients with ACS, our population had higher rates of ID. Zeller et al reported a frequency of 29% in a population of 836 patients with ACS who underwent cardiac catheterization (a retrospective subgroup analysis of patients with ACS from the AtheroGene cohort study),^21^ and Silva et al described a prevalence of 36% in 817 patients admitted with ACS.^1^ However, a recent meta-analysis that included seven cohort studies (generally of small dimension and with high heterogeneity) of patients with ACS who were stratified by ID status showed an overall ID prevalence of 43%,^24^ very similar to our data. Interestingly, in a cohort of elderly patients (mean age 78 years) with ACS, ID was even more frequent, present in 60% of cases.^22^

Patients with ID were more often female and were more frequently treated with beta-blockers and insulin. These patients were admitted more commonly with NSTEMI rather than STEMI, which was also a feature of the population studied by Martins et al.^31^

Regarding adverse events during up to a 35-month follow-up, there were significant differences in emergency department admissions, more frequent in the group with ID. There was no impact of ID on stroke/TIA, bleeding events, ACS, or CV/all-cause mortality during follow-up.

Of note, patients with ACS in our cohort were not treated nor discharged with potent P2Y12 inhibitor prasugrel, as recommended by recent ESC ACS guidelines (class IIa recommendation of prasugrel in favor of ticagrelor),^32^ as it is not available in our center.

Silva et al^1^ reported ID as a predictor of a composite of all-cause mortality and severe HF in a cohort of patients admitted with ACS. Gonzalez-D'Gregorio et al found a trend toward significance regarding mortality for patients with ID during a median follow-up of 5 years while Zeller et al reported ID as a predictor of MACE defined as myocardial infarction and CV mortality during a mean follow-up of 4 years.^21,22^

Finally, we analyzed the role of different factors in patients' prognosis. All-cause death was predicted by age, presence of anemia, and NT-proBNP levels while ID had no impact on none of the evaluated events. In an international pooled cohort of 1506 patients with chronic HF, ID was a predictor of mortality independent of other already known predictors (namely anemia); while ID still predicted outcomes in patients without anemia, the same was not true in anemic patients without ID.^13^ Unfortunately, data on the etiology of anemia were not available in this cohort, which would be interesting to analyze, as 31% of those patients who did not have ID also presented with anemia.

Anemia has been known to be associated with worse outcomes in patients with HF,^14^ and there is plenty of evidence of its negative prognostic impact on patients with ACS, while also being related to worse outcomes regarding HF events in this population.^33–35^ As iron is a key mitochondrial enzymatic cofactor and is thus essential in cellular oxygen metabolism, it is hypothesized that, because mitochondrial dysfunction leads to myocardial ischemia–reperfusion injury, ID may exacerbate myocardial damage in ACS.^36,37^ There is, however, one small study that, paradoxically, found that patients with ID had a better in-hospital outcome, which seemed to be associated with a smaller myocardial reperfusion injury; the mechanisms underlying this discovery were unclear.^37^

The role of anemia in the acute setting of an ACS is likely to have a greater impact regarding mortality because of the reduction in myocardial oxygen supply together with increased oxygen demand, prolonging and exacerbating ischemia.^38,39^ The inflammation state present in ACS could promote functional ID, which could be associated with some degree of metabolic dysfunction at the level of the myocardium, promoting HF.^2,5,33^

## Limitations

This is a one-center, retrospective study, with all the limitations that are inherently associated. Patient data were collected through admission and discharge notes that could lack information regarding history and associated comorbidities. Of note, LVEF's specific value at admission was not always described in the clinical notes, and it was not routinely assessed at discharge. Data on mechanical ventilation were not available in all patients. Furthermore, there were no serial iron status measurements during the course of the index hospitalization nor on follow-up, which would be warranted on further studies on this matter. In addition, there were very limited data regarding treatment of ID during follow-up, and cause of death was not available in most of the cases. A minority of patients were lost to follow-up because of transfer to other institutions and inability to access the medical records of the patients who died. Finally, there were not sufficient cases to analyze subgroups of functional/absolute ID, or with and without anemia.

## Conclusion

There are still scarce data in the literature concerning ID in patients with ACS. Our study shows that ID is prevalent in this population, affecting almost half of the patients with ACS. Anemia seems to have a greater impact on mortality in the setting of an ACS. Further studies with larger numbers are required for more definite conclusions, to evaluate the impact of functional/absolute ID, with or without anemia, on promoting HF, and to investigate the potential prognostic role of iron supplementation in patients with ACS and ID.
